# A Contribution to Surgery from Its Youngest Specialty, Dental Surgery.—The Surgical Engine

**Published:** 1894-09

**Authors:** W. G. A. Bonwill

**Affiliations:** Philadelphia, Pa.


### SELECTIONS.

### A Contribution to Surgery from its Youngest Specialty, Dental Surgery.—The Surgical Engine.

Read in the Section on Surgery and Anatomy at the Forty-fifth Annual Meeting of the American Medical Association, held at San Francisco, June 5-8, 1894.

BY W. G. A. BONWILL, D.D.S., PHILADELPHIA, PA.

While every medical man has heard and read of this instrument for the past twenty years, but few have ever witnessed the application of it in the surgery of the bones and tissues. Outside of Philadelphia—my home—it has been but little used, so slow are surgeons to take hold of new appliances, and particularly this engine, which, to so many, seems so formidable that, while they can not doubt its great efficacy yet they fear to handle an instrument driven at the high speed of from one to twenty thousand revolutions a minute.

The dental engine had been tried for minor operations and was better than nothing; yet it was not until this duplicature of the human arm and hand was developed, that it was possible to call it a truly surgical engine.

This I present you to-day, is the only type that is of any value and is based on the surest basal principles of mechanics. It is not necessary to describe it in detail. Suffice it to say that a look at its working parts will satisfy the most skeptical that it must do all that is claimed for it.

As a further guarantee of its value in surgery, I need but mention a few of those eminent in this line. Both the elder and younger Gross, Agnew, of Philadelphia, and Horsley, of London. These are enough.

Be it said to the credit of the conservatism of surgeons, it would have been more largely used had I not parted with it and sold to a manufacturing firm, hoping to have it more extensively made and better and cheaper. But I made a great mistake. It is not too late to have it remedied. As you see it here in operation there is every element about it calculated to perform any operation in surgery on soft or hard tissues, and it is so simple any one can take care of it, and so cheap that any surgeon of extensive practice can afford to have one. Beside, it is so made that it is used for a dental engine and wherever it is to be found, in any town or hamlet, in the possession of a dentist, it can be borrowed at any time and made available for surgical work by simply adding to it a crank to give it higher speed; so that an amicable arrangement can be made with the dentists for its use, and they would be too glad to be of service in assisting the surgeon anywhere.

In this way the first cost to the surgeon is nothing, further than having a few instruments adapted for its special work which would be but a trifle. Then its universal presence could be relied upon at any moment and anywhere if it is in the possession of a dentist. This one item, of combining the dental with the surgical power, must militate largely in its favor and one's surroundings can be adapted to its use. This argument is no mean one. While not so good for the manufacturer, yet to the *user* of it a favor not to be resented.

Up to the time of the death of both of the Professors Gross, not an operation on the bones was performed but I did it for them.

As to Professor Horsley, of London, he said before the Washington Congress: "Thanks to one of your fellow-countrymen—Dr. Bonwill. Without this engine I would not like to undertake the formidable operations I have to perform on the skull for removal of tumors and treatment of nervous affections."

Its range enables you to perform the most delicate as well as the most extensive operations, and can be confined to 100 revolutions or run up into the 1,000 revolutions.

It will remove in a few minutes the hardest osseous tumor, or with the diamond will cut a stone in the bladder into atoms.

In plastic surgery wherever tissues are to be united there is no loss of substance and success is more certain. In the bones, whether taking away the whole shaft leaving nothing but the periosteum for renewal of the parts—there is no necessity for opening into the bone further than merely exposing for access, thereby saving the severing of important arteries or interfering with nerve tissue. In the reduction of disunited fractures and the absolute apposition with which they are afterwards held by special appliances, it is of great value.

It can be used in fractures of the petella without opening into the membranes. Removal of the whole of the superior or inferior maxilla without other access than by the mouth. Its aid with its special appliances enables you in fractures of the skull to elevate the parts without trephining.

Removal of ths bones of the coccyx has been done in a few minutes and no injury done to the rectum or shock to the surroundings. With a special instrument I have, the vault of the cranium can be entered at any point without a trephine, and of any shape, without any danger of injury to the dura mater or much loss of bone.

In operations upon the cervix, the diamond wheels it carries at high speed chafe off the hardened tissues with but trifling loss, and enable the parts to more certainly be brought in apposition, and left nearly the normal size of cervix.

It is unnecessary to speak further of its possibilities, as the ingenious surgeon would soon see that it would be his constant companion and when once he had learned to manipulate it, it would be as much of a *sine qua non* as it is to-day a part of modern dental surgery.

No one can estimate the value alone in time saved in all cases and, from its high speed and capability of reaching 20,000 revolutions a minute, *pain* itself can almost or quite be annihilated or prevented, and if nothing more the shock is comparatively light, the effort at healing by first intention is made certain in every case, and from the smoothness of the bone cut, drilled, burred or sawed, there are no spiculæ of bone left to induce the least inflammation or necrosis.—*The Journal of the American Medical Association.*

